# Engineering *Yarrowia lipolytica* to produce biodiesel from raw starch

**DOI:** 10.1186/s13068-015-0335-7

**Published:** 2015-09-15

**Authors:** Rodrigo Ledesma-Amaro, Thierry Dulermo, Jean Marc Nicaud

**Affiliations:** INRA, UMR1319 Micalis, 78350 Jouy-en-Josas, France; AgroParisTech, UMR Micalis, Jouy-en-Josas, France; Institut Micalis, INRA-AgroParisTech, UMR1319, Team BIMLip, Biologie Intégrative du Métabolisme Lipidique, CBAI, 78850 Thiverval-Grignon, France

**Keywords:** *Yarrowia lipolytica*, Consolidated bioprocess, Starch, Metabolic engineering, Biodiesel

## Abstract

**Background:**

In the last year, the worldwide concern about the abuse of fossil fuels and the seeking for alternatives sources to produce energy have found microbial oils has potential candidates for diesel substitutes. *Yarrowia lipolytica* has emerged as a paradigm organism for the production of bio-lipids in white biotechnology. It accumulates high amounts of lipids from glucose as sole carbon sources. Nonetheless, to lower the cost of microbial oil production and rival plant-based fuels, the use of raw and waste materials as fermentation substrate is required. Starch is one of the most abundant carbohydrates in nature and it is constituted by glucose monomers. *Y. lipolytica* lacks the capacity to breakdown this polymer and thus expensive enzymatic and/or physical pre-treatments are needed.

**Results:**

In this work, we express heterologous alpha-amylase and glucoamylase enzymes in *Y. lipolytica*. The modified strains were able to produce and secrete high amounts of active form of both proteins in the culture media. These strains were able to grow on starch as sole carbon source and produce certain amount of lipids. Thereafter, we expressed both enzymes in an engineered strain able to overaccumulate lipids. This strain was able to produce up to 21 % of DCW as fatty acids from soluble starch, 5.7 times more than the modified strain in the wild-type background. Media optimization to increase the *C*/*N* ratio to 90 increased total lipid content up to 27 % of DCW. We also tested these strains in industrial raw starch as a proof of concept of the feasibility of the consolidated bioprocess. Lipid production from raw starch was further enhanced by the expression of a second copy of each enzyme. Finally, we determined in silico that the properties of a biodiesel produced by this strain from raw starch would fit the established standards.

**Conclusions:**

In this work, we performed a strain engineering approach to obtain a consolidated bioprocess to directly produce biolipids from raw starch. Additionally, we proved that lipid production from starch can be enhanced by both metabolic engineering and culture condition optimization, setting up the basis for further studies.

**Electronic supplementary material:**

The online version of this article (doi:10.1186/s13068-015-0335-7) contains supplementary material, which is available to authorized users.

## Background

The excessive use of petroleum sources cannot cope with the increasing worldwide energy consumption and the environmental requirements to prevent global warming. Bio-lipids produced either by plant or microorganisms are one of the most promising sustainable and renewable alternative sources of energy [1]. Microbial oils present several advantages over plant-based oils: they (1) sometimes can be synthesized from waste products as carbon source (such as glycerol or lignocellulosic materials), (2) they are independent of climate and seasonal conditions, (3) they have short process cycles, (4) industrial-scale fermentations can be rapidly adapted to market needs, (5) the land area needed for microbial cultivation is much smaller than that required for plant growth and importantly (6) microorganisms can be modified by metabolic engineering approaches, which can be utilized to enrich specific desired fatty acids within the microbial oils [2]. However, there are also drawbacks in the use of microbial oils mostly associated to the production costs. The requirement of a controlled environment of aeration, temperature and agitation, the need for aseptic conditions and the recovery of the oil from the cells increase the cost of the whole process. On the one hand, it is important to reduce the fermentation costs by maximizing the yield and productivity. It is essential for the economical viability of the processes to optimize the culture conditions and enhance the fermentation capacity by strain engineering. On the other hand, it is recommended to lower the cost of the substrates present in the culture media. In this regard, glucans are the most abundant polymer in plant biomass, with cellulose as the major structural component and starch as the major energy reserve [3].

Starch is a polymer of glucose formed by amylopectin (α-1,4-d-glucopyranose chains with α-1,6 branching points) and amylose chain helix (α-1,4-linked d-glucopyranose units). It is an import food and feed source and a preferred substrate for the production of many industrial products. In fact, the use of starch to produce biofuels, such as bioethanol, is already a consolidated technology [4]. The general process in the industrial fermentations begins with the obtaining of the starch slurry after milling the raw starch. The starch slurry will be used as a substrate for liquefaction at high temperature where thermostable alpha-amylases are added to the mixture to produce oligosaccharides. Thereafter, the broth is cooled down and glucoamylases are added to release glucose and maltose in the saccharification process. Finally, the glucose-enriched mixture is used for the fermentation. Usually, saccharification and fermentation steps take place together in the so-called simultaneous saccharification and fermentation (SSF). A cost-effective procedure requires the consolidated bioprocessing (CBP) by a single organism that accomplishes liquefaction, hydrolysis and fermentation. Unfortunately, often those organisms able to degrade raw starch are not good enough in the fermentation of the desired product. An illustrative example is the case of ethanol production where over 150 amylolytic yeast strains have been reported to be impractical in industrial use because of limited characteristics [5]. The alternative proposed approach was to convert *Saccharomyces cerevisiae* into amylolytic yeast. Therefore, many different amylases have been expressed in baker yeast to make it able to produce ethanol from starch in CBP manner [3, 6]. The combination of α-amylases and glucoamylases has been considered as minimum requirement for the complete hydrolysis of raw starch [6].

*Yarrowia lipolytica* is well-known oleaginous organism proven suitable for many different industrial processes. It is a safe organism [7] widely used to produce food grade products such as organic acids, polyalcohols, aromas, emulsifiers, surfactants and proteins [8]. Moreover, during the last years it has been a model organism for biofuel production, especially for those derived from fatty acids [9–11]. Additionally, *Y. lipolytica* is suitable for metabolic engineering approaches since there is a wide range of molecular tools to manipulate it [12, 13], a well-curated genome available [14], its metabolism has been studied in detail and two genome scale metabolic model exist [15, 16]. Additionally, several works have analyzed it from a systems biology point of view using different omics data (metabolomics, proteomics, transcriptomics and fluxomics) [17–20], which all together enable systems metabolic engineering of this organism.

So far, metabolic engineering has already boosted lipid production in this yeast. Different target genes for overexpressions and deletions have been identified and manipulated to increase total fatty acid content. As an example, our group found that blocking beta-oxidation by deletion of the six *POX* genes [21] or the *MFE* gene [22] and overexpression of enzymes leading to TAG production, such as *DGA2* [23] and *GPD1* [22], enhanced lipid production. Recently a modified strain was able to reach a very high carbon to lipid conversion yield (84.7 % of theoretical maximal yield) and very high lipid titers (~55 g/L) under optimized conditions, supporting the feasibility of *Y. lipolytica* to produce biodiesel [24]. Nonetheless, as discussed above, it is preferred to use inexpensive raw materials such as starch or lignocelluloses instead of glucose as carbon sources in the fermentations. Unfortunately, *Y. lipolytica* is not able to degrade either cellulose or starch. A recent work by Wei et al. [25] has modified this oleaginous organism by the heterologous expression of cellulases to make it able to utilize cellulosic substrates. However, no work has yet reported the use of starch by *Y. lipolytica*. Nonetheless, two alpha-amylases—one of the enzymes required for degrading starch—have been expressed in this host [26, 27]. The aim of these works was protein expression and purification only and there are no reports about the capacity of these strains to grow on raw starch.

Here, we engineer *Y. lipolytica* to consume starch and produce lipids. For this purpose, we expressed two heterologous enzymes, one alpha-amylase and one glucoamylase from rice and *Aspergillus*, respectively. On the one hand, we chose alpha-amylase from rice since it has been previously expressed and secreted successfully in *Y. lipolytica* [27] and, on the other hand, we chose glucoamylase from *Aspergillus niger* which is widely used by the industry [28]. Both enzymes were successfully secreted to the medium in an active form. Therefore, the strain overexpressing both proteins was able to grow on starch as sole carbon source. To enhance lipid production from starch, we introduced these two genes into a previously engineered strain with increased fatty acid synthesis capacity and blocked for beta-oxidation. The final strain was able to produce high amounts of lipids from starch. To prove the feasibility of the consolidated bioprocess, we grow our engineered strain in industrial raw starch and evaluate lipid production and composition. Additionally, a second copy of each gene further boosted total lipid production showing besides a fatty acid profile suitable for a biodiesel.

## Results and discussion

### The heterologous expression of alpha-amylase from *Oryza sativa* makes *Y. lipolytica* able to degrade starch

**Fig. 1:**
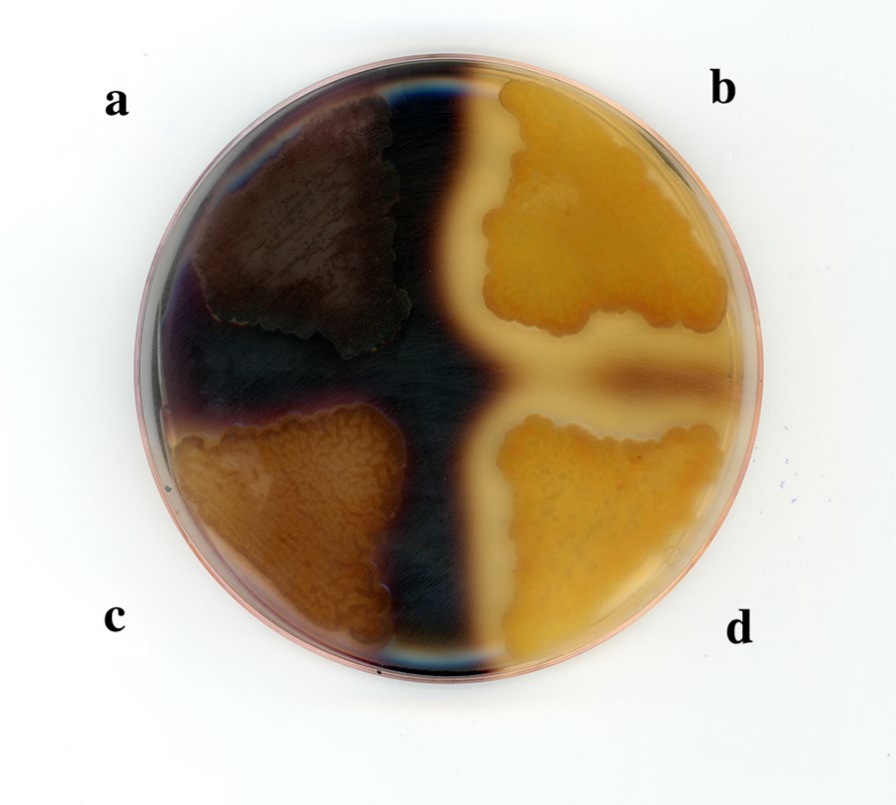
Starch-containing YPD plate. YPD plate containing starch after 3 days of incubation at 28 °C. The plate was stained with iodine vapor. The strains able to clarify starch were distinguished by the clear zone around the colonies. *A* the wild type (JMY2900), *B* expression of alpha-amylase (JMY5077), *C* expression of glucoamylase (JMY5083) and *D* expression of alpha-amylase and glucoamylase (JMY5017)

**Fig. 2:**
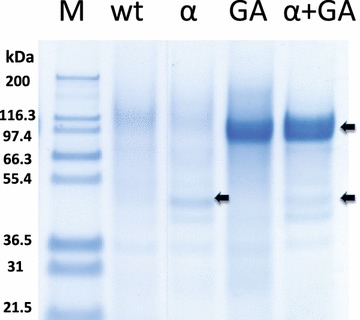
SDS-PAGE gel of the culture supernatant. Proteins present in the supernatant of the culture from *wt* (wild type, JMY2900), *α* (strain expressing alpha-amylase, JMY5077), *GA* (strain expressing glucoamylase, JMY5083) and *α* *+* *GA* (strain expressing alpha-amylase and *GA*, JMY5017). *M lane* shows the molecular marker. *Arrows* indicate the bands corresponding to the expected sizes of the expressed proteins

α-Amylase is one of the two minimal activities required to completely degrade raw starch [6]. In this work, we overexpressed and secreted the α-amylase of *Oryza sativa* in *Y. lipolytica* strain JMY5077, which has been previously actively produced in this yeast [27]. Contrary to Park et al. [27], we expressed a codon-optimized α-amylase gene under the control of the strong and constitutive TEF promoter [29]. Additionally, we substituted its native signal peptide by the pre-signal sequence of the main extracellular lipase, Lip2p, followed by three X-Ala motifs (see Additional file 1: Table S1) [30]. The generated strain, overexpressing the rice α-amylase, was able to produce the active enzyme according to the clear zones around the colonies on starch-containing YPD plates (Fig. 1b), contrary to the wild type (Fig. 1a). In addition, the supernatant of a glucose-based culture showed two bands on acrylamide gel corresponding to the expected sizes of the two different processed variants of the protein, 45 and 47 kDa (Fig. 2), as it has been previously described [27]. The presence of the protein in the supernatant further supports the correct secretion of the enzyme. This supernatant was able to generate clear zones after applying to a starch-containing plate indicating the secretion of an active form of the protein (Additional file 2: Figure S1).

**Fig. 3:**
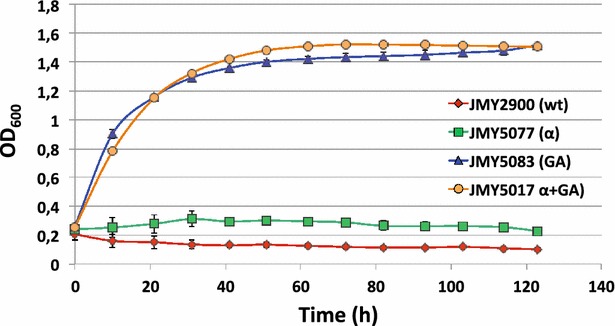
Growth curve in starch media as sole carbon source. Growth curve obtained after measuring the OD_600_ in 96 well plates. *wt* (wild type, JMY2900), *α* (strain expressing alpha-amylase, JMY5077), *GA* (strain expressing glucoamylase, JMY5083) and *α* *+* *GA* (strain expressing alpha-amylase and *GA*, JMY5017). Showed values are the average of three independent experiments

**Fig. 4:**
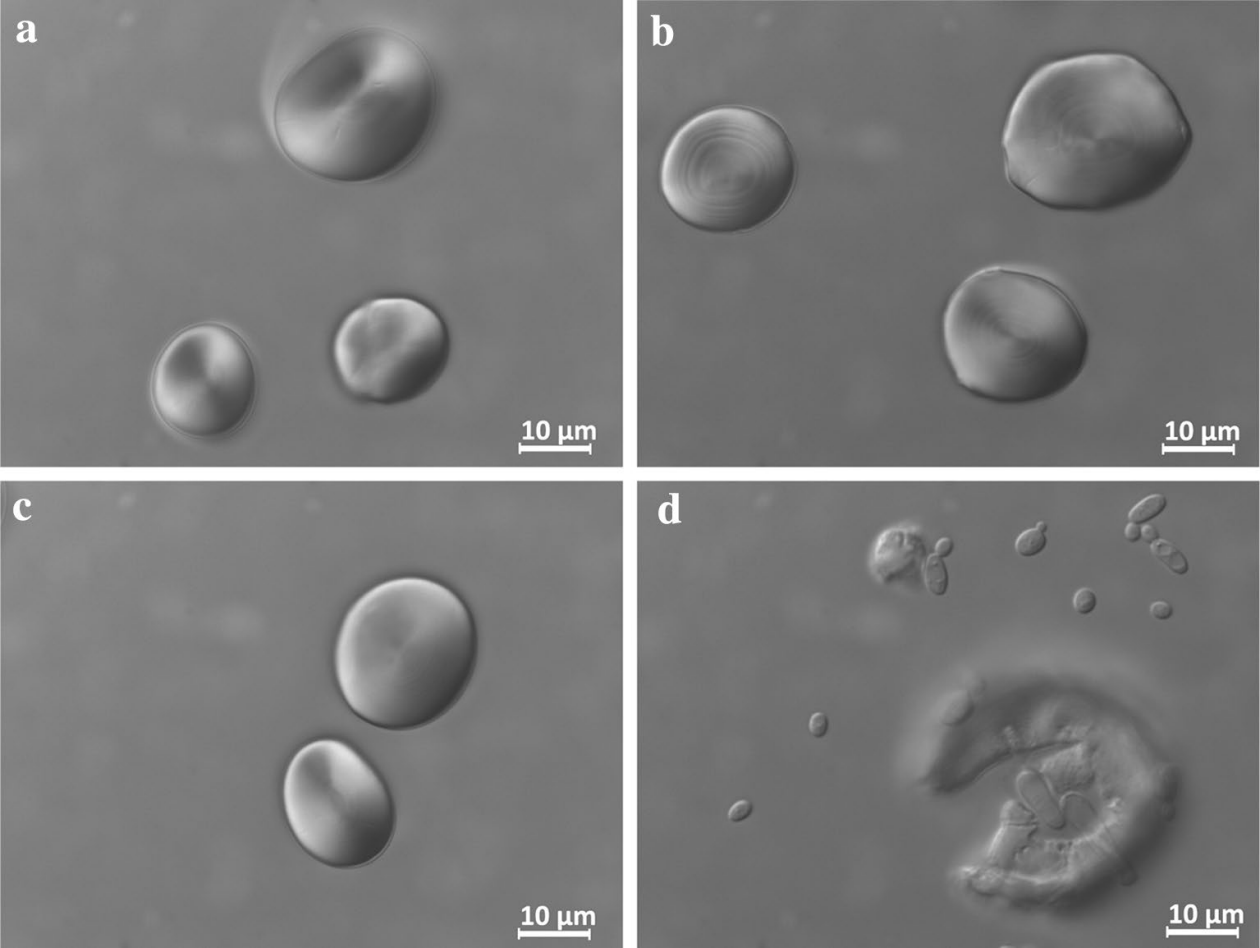
Optical microscope images of the granules of raw starch incubated with different *Y. lipolytica* strains. **a** The wild type (JMY2900), **b** expression of alpha-amylase (JMY5077), **c** expression of glucoamylase (JMY5083) and **d** expression of alpha-amylase and glucoamylase (JMY5017)

Despite the proved expression and secretion of the active α-amylase, the modified strain was unable to grow on starch-based medium with no other carbon source (Figs. 3, 4). Cellular growth was followed either in soluble starch by the OD_600_ measurement in liquid media containing soluble starch (SS) (Fig. 3) or in raw starch by the presence of yeast cells under optical microscope (Fig. 4). These results can be explained because α-amylases hydrolyze the internal α-1,4-bonds of amylose and amylopectin at random, producing mainly maltodextrins with a length of 10–20 glucose residues, that *Y. lipolytica* cannot assimilate. Although these enzymes can also release maltose and free glucose, these may not be enough to allow efficient growth. Therefore, additional enzymatic activities to release glucose units are required to use *Y. lipolytica* as consolidated bioprocess using starch.

### The expression of glucoamylase from *Aspergillus niger* in *Y. lipolytica* is sufficient to allow growth on soluble starch

Glucoamylase is recognized as the most important enzyme, which is responsible for the progressive hydrolysis of starch from non-reducing ends to release β-d-glucose units and saccharification of the polymers [3]. Many glucoamylases are currently used by the industry and one of the most important is one from *A. niger* [28]. Additionally, successful metabolic engineering approaches to produce ethanol in *S. cerevisiae* using starch as sole carbon source are based in the single heterologous expression of glucoamylase [31–33]. Also, the production of glucoamylase from *Aspergillus* was essential in the saccharification step prior to a fermentation to produce lipids using *Lipomyces starkeyi* [34]. Glucoamylase was expressed in *Y. lipolytica* wild-type strain (Po1d) generating the strain JMY5083. Glucoamylase signal sequence was replaced by the targeting sequence of Lip2 and its expression was controlled by the promoter TEF (Additional file 1: Table S1). As expected, the modified strain of *Y. lipolytica* showed smaller clear zones around the colonies comparing to the α-amylase expression on YPD plates containing starch (Fig. 1c). This is due to the fact that iodine staining decreased with the length of the amylase chain and therefore it is preferentially used for identifying alpha-amylase activity, which release shorter chains of amylase. Nonetheless, if glucoamylase activity is very high this can also show clarification zones, as we observed [35]. A band corresponding to the expected size of the protein, 68.2 kDa, was found in the supernatant of the culture of the engineered strain in glucose-based media (Fig. 2), proving efficient expression and secretion of the *Aspergillus* enzyme in *Yarrowia*.

Interestingly, growth of the glucoamylase expressing strain was similar in a media containing soluble starch (SS) (Fig. 3) and in media containing glucose as sole carbon source, thus indicating an efficient release of glucose units by the active glucoamylase.

Nonetheless, this strain was unable to grow in raw starch medium (RS). This result may be explained due to a lack of α-amylase activity (Fig. 4) which may be required to facilitate the accessibility of the glucoamylase to this substrate [36].

### *Y. lipolytica* co-expressing alpha-amylase and glucoamylase produces lipids from starch

We showed that *Y. lipolytica* expressing α-amylase is able to clarify raw starch and how the strain expressing glucoamylase can release enough glucose from soluble starch to permit growth. Nonetheless, the single expression of one of the amylases is not enough to make Yarrowia able to use raw starch as carbon source. Therefore, we decided to combine the two enzymes in the same strain. In addition, synergistic effects have been found in the co-expression of both enzymes as well as higher titers in biomass and ethanol production from starch in baker yeast [37–39]. As expected, the constructed strain expressing both enzymes, JMY5017, was able to generate clear halos when growing in YPD containing starch (Fig. 1d), indicating α-amylase activity. Additionally, we found bands corresponding to the expected size of both proteins in the supernatant of a glucose-based culture, suggesting their expression and secretion (Fig. 2). This strain was able to grow in soluble starch as sole carbon source as good as the strain expressing the glucoamylase alone (Fig. 3), since no statistical significance was found when comparing either growth rate or final biomass between JMY5017 and JMY5083. In this case, JMY5017 was able to degrade the granules of raw starch and release enough glucose units to permit the yeast growth (Fig. 4) contrary to the strains producing a single enzyme.

**Fig. 5:**
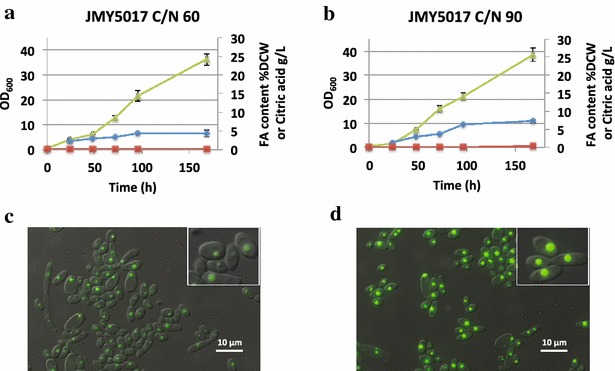
Lipid production in wild-type background. **a**, **b** Show the strain expressing alpha-amylase and glucoamylase in the wild-type background; the growth in *green* (OD_600_), the percentage of fatty acids in the DCW (%FA) in *blue* and the citric acid produced (g/L) in *red*. Two culture media were used as described in “Methods” with different amounts of starch; YNBCN60 (*C*/*N* 60) and YNBCN90 (*C*/*N* 90). **c**, **d** Show fluorescence microscopy images where the lipid bodies were stained with Bodipy. **c** Corresponds to the experiment A, while **d** corresponds to the experiment B. All presented data are the average of at least two independent experiments. The *panels* show representative cells that have been enlarged (×2)

Due to the interest in producing bio-based alternatives to petroleum sources from renewable materials, we further wanted to investigate the ability of our modified strain to produce bio-lipids from starch (SS). We therefore make cultures in two different media with high *C*/*N* ratio, which is known to trigger lipid accumulation in this yeast [40]. The modified strain co-expressing the two enzymes produces up to 4.4 ± 0.9 % of the DCW as fatty acids in the culture media with soluble starch and a *C*/*N* ratio of 60. When the *C*/*N* ratio was increased to 90, the same strain doubles its lipid accumulation capacity, reaching 7.2 ± 0.4 % of the DCW as fatty acids (Fig. 5a, b). In both culture media, this strain consumed 49.4 ± 2.4 % of the starch after 6 days of culture. In both YNBC/N 60 and YNBC/N90 similar final biomass was reached 11.33 ± 0.17 and 11.14 ± 0.13 g/L, respectively. Accordingly, total lipid production was 0.49 ± 0.09 and 0.80 ± 0.06 g/L, respectively. This experiment suggests that starch can be used for lipid production in this strain and that media composition and culture condition optimization can further enhance lipid accumulation. Additionally and according to fluorescence microscopy images, the fatty acids are mainly accumulated in lipids bodies (Fig. 5c, d) as it normally occurs under nitrogen limitation conditions.

### Lipid overproducer strain of *Y. lipolytica* produces high amounts of fatty acids from starch

We have previously generated an engineered strain of *Y. lipolytica* containing multiple modifications able to accumulate high amounts of lipids, JMY3501 [41]. This strain has been blocked for the β-oxidation by the deletion of the six *POX* genes, avoiding lipid degradation in the peroxisome [21]. It has been also blocked for *TGL4*, the main intracellular lipase in charge of the release of fatty acids from TAGs stored in the lipid body [42]. Additionally, it overexpresses the gene *GPD1* [22] involved in the glycerol-3-phosphate formation, a precursor of the TAG, and the gene *DGA2* [23], encoding an acyltransferase involved in the last step of TAG formation. We thus further modified this strain by overexpressing the α-amylase and glucoamylase genes to generate the strain JMY5035.

**Fig. 6:**
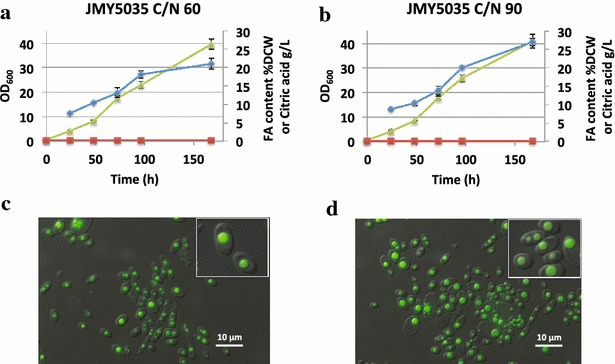
Lipid production in lipid overproducer background. **a**, **b** Show the strain expressing alpha-amylase and glucoamylase in the lipid accumulating background JMY3820; the growth in *purple* (OD_600_), the percentage of fatty acids in the DCW (%FA) in *blue* and the citric acid produced (g/L) in *green*. Two culture media were used as described in “Methods”; YNBCN60 (*C*/*N* 60) and YNBCN90 (*C*/*N* 90). **c**, **d** Show fluorescence microscopy images where the lipid bodies were stained with Bodipy. **c** Corresponds to the experiment A while **d** corresponds to the experiment B. All presented data are the average of at least two independent experiments. The *panels* show representative cells that have been enlarged (×2)

Lipid content of JMY5035 was compared when it grew in soluble starch-based medium (SS) presenting different *C*/*N* ratios (60 and 90). As we expected, JMY5035 strain greatly improved lipid content, up to 21.1 ± 1.4 % of the DCW, 5.7 times more than JMY5017 (Fig. 6a, c). Total lipid was further enhanced when a *C*/*N* ratio of 90 was used, where JMY5035 reached 27.0 ± 1.4 % of DCW as fatty acids (Fig. 6b, d). These results are in accordance with previously publish data of the parental strain JMY3501, which after growing in glucose *C*/*N* ratio of 60 accumulated up to 25 % of DCW as fatty acids [41]. Similar biomass was formed in both *C*/*N* ratios; 11.55 ± 0.07 and 12.36 ± 0.16 g/L for *C*/*N* 60 and 90, respectively. Accordingly, total lipid production was 2.44 ± 0.15 and 3.34 ± 0.13 g/L, respectively. At the end of the culture, the strain JMY5035 was able to consume 60.3 ± 6.4 % of the starch added to the media. Interestingly, citric acid amounts produced in the conditions tested were very low, which is preferred in the industrial production of lipids using *Y. lipolytica*. Both slower growth and no citric acid production can be explained by the lack of the overflow through the glycolysis pathway, which would be limited by the rate of glucose release from starch.

Therefore, we here not only proved that our modified *Y. lipolytica* strains can accumulate lipids from starch but also that the amount of produced lipids can be enhanced by both optimization of culture conditions and strain engineering.

### Engineered *Y. lipolytica* strains produce biolipids suitable for biodiesel from industrial raw starch

**Fig. 7:**
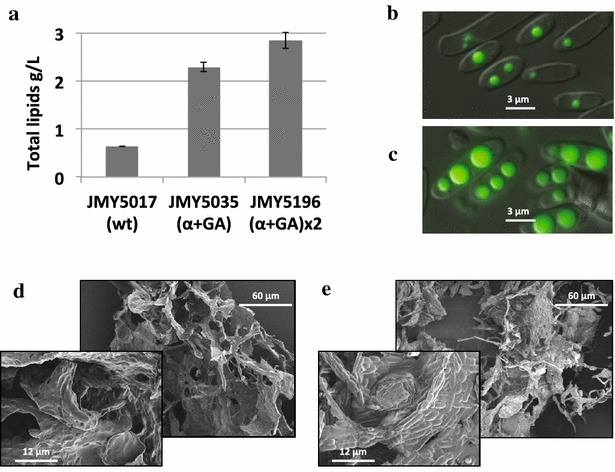
Flask fermentation to produce lipids from industrial raw starch. **a** Shows the g/L of lipids produced when the strains were grown in industrial starch media (IS). The differences between the three strains are statistically significant among each other (*p* < 0.05). **b**, **c** show fluorescence microscopy images where the lipid bodies were stained with Bodipy. **b** Correlates with the strain wt (α + GA), JMY5017, and **c** with the strain 3820 (α + GA) × 2, JMY5196. Industrial starch under the electron microscope **d** when no cells are present in the solution and **e** after growing the cells of JMY5196

To complete our proof of concept about *Y. lipolytica* as a consolidated bioprocess to produce lipids from starch, we wanted to test our strains in an industrial starch media (IS). For this purpose, we obtained starch from the company Tereos Syral and we used it directly as a substrate for flask fermentation. To cope with the higher amounts of substrates normally used in large-scale fermentations, we constructed additional strains expressing one extra copy of each of the enzymes (JMY5196) using JMY5035 as parental strain. These strains were able to grow and produce lipids using the industrial product containing starch as carbon source (IS media) in flask under non-optimized conditions (Fig. 7). The wild-type background overexpressing the two enzymes (JMY5017) produces 0.64 ± 0.08 g/L of lipids, while the same overexpressions in the engineered background for lipid accumulation (JMY5035) reached 2.29 ± 0.94 from 22.5 g/L of glucose equivalents in the substrate.

In addition, we proved that a second copy of both α-amylase and glucoamylase further increase lipid production up to 2.84 ± 0.16 g/L, which represent a yield *Y*_FA/S_ = 0.13 ± 0.07 g/g (grams of fatty acids produced per gram of glucose equivalents in the industrial starch added to the media). The parental strain JMY3501 produced similar lipid titers, 3.2 g/L from 60 g/L of glucose, and therefore with a lower yield *Y*_FA/S_ = 0.053 g/g [41]. Although the total lipid titer remains low compared to other engineered strains in bioreactor optimized conditions [24], here we set up a proof of concept that can be easily transferred to different genetic backgrounds and cultured in optimized large-scale conditions.

Surprisingly, we found those strains able to grow in the industrial starch on the surface of the insoluble fractions (coming from wheat cells) present in this substrate, as revealed in the electronic microscopy [Fig. 7d (control with no cells), e (attached cells)]. These preliminary data could suggest the ability of *Y. lipolytica* to bind plant biomass, which could be useful in cell immobilization approaches. Additionally, this interesting feature could be exploited towards the use of lignocellulosic material using this yeast. This fact is in accordance to previous reports that show the ability of *Yarrowia* to bind to hydrophobic substrates in an inducible manner [43] due to Lewis acid–base interactions rather than hydrophobic properties of the cell surface [44] Nonetheless, more experiments must be performed to confirm these results, which are currently undergoing.

As we previously discussed, microbial lipids can be used as biodiesel after transmethylation reaction to form fatty acids methyl esters (FAMEs) [45]. It is important to notice that the fatty acid profile (differences in carbon chain length and number of double bonds) of the biolipid has a strong influence in the biodiesel quality [46]. Therefore, we analyzed the fatty acid composition of our strains after growing in industrial starch (Additional file 3: Figure S2). As expected, most abundant fatty acids are 18:1, 16:0, 18:2, 16:1 and 18:0. Nonetheless, significantly differences were found between the wild-type background and the lipid accumulating background, where 16:0 was two times higher and 18:1 was diminished in 20 %. We decided to further analyze the theoretical application of the FAMEs from our engineered strains for the production of biodiesel. Several biodiesel quality standards have been established in different countries, including the USA (ASTM D 6751) and Europe [EN 14214 (vehicle use) and EN14213 (heating oil)]. Here, the theoretical properties of *Y. lipolytica* biolipids for biodiesel production were analyzed according to the most restrictive standard, EN 14214. We therefore evaluated the following parameters (Additional file 4: Table S2): (a) kinematic viscosity at 40 °C, (b) density at 15 °C, (c) iodine value, (d) heating value, (e) cetane number and (f) cold filter plugging point. Interestingly, all biolipids from the tested strains fit the required standard values, presenting thus, a promising composition for being used as biodiesel.

## Conclusion

In summary, we successfully expressed and secreted heterologous amylases in *Y.* *lipolytica*, α-amylase from *O. sativa* and glucoamylase from *A. niger*. To our knowledge this is the first report on the expression of an active glucoamylase in *Yarrowia*. The modified strain was able to grow in starch as sole carbon source. Additionally, we performed the same expressions in a lipid accumulating background and it proves to accumulate high amounts of lipids. Interestingly, the amount of intracellular lipid was not only dependent in the genetic background but also in the *C*/*N* ratio of the media, which proof the potential enhancement of the yield and productivity of the process after culture optimization at bioreactor scale. Finally, the engineered strain was able to produce lipids from industrial raw starch what is a proof of concept in the use of *Y. lipolytica* as a consolidated bioprocess to generate biodiesel. Interestingly, the theoretical analysis of the biodiesel properties of the FAMEs generated from starch fits the most restrictive regulatory standard.

## Methods

### Strains and media

The *Y. lipolytica* strains used in this study were derived from the wild-type *Y. lipolytica* W29 (ATCC20460) strain. All of the strains used in this study are listed in Table 1. Media and growth conditions for *Escherichia coli* and *Y.* *lipolytica* have been described elsewhere [47]. Minimal medium (YNBCN60) contained 0.17 % (wt/vol) yeast nitrogen base (YNBww), 6 % glucose (wt/vol; Merck, Fontenay-sous-Bois Cedex, France) 0.15 % (wt/vol) NH_4_Cl and 50 mM phosphate buffer (pH 6.8). Soluble starch medium (SS) was similar to YNBCN60 but substituting glucose for soluble starch, 1 % in the case of microplate assay (see results), 6 % in C/N60 and 9 % in C/N90, all of them contained 0.17 % (wt/vol) yeast nitrogen base (YNBww), 0.15 % (wt/vol) NH_4_Cl and 50 mM phosphate buffer (pH 6.8). Raw starch medium (RS) was similar to YNBCN60 by substituting glucose for wheat raw starch (1 %) (Sigma-Aldrich), and as YNBCN60, 0.17 % (wt/vol) yeast nitrogen base (YNBww), 0.15 % (wt/vol) NH_4_Cl and 50 mM phosphate buffer (pH 6.8). The industrial starch medium (IS) was similar to YNBCN60 but substituting glucose for 25 % industrial product containing starch (DZ starch) provided by Tereos Syral (Belgium).

**Table 1:** Strains used in this work

Strains	Genotype or other relevant characteristics	Source or reference
*E. coli*		
DH5α	*Φ80dlacZΔm15,* *recA1,* *endA1,* *gyrA96,* *thi*-*1,* *hsdR17 (r*_*k*_−*, m*_*k*_+*),* *supE44,* *relA1,* *deoR, Δ(lacZYA*-*argF)U169*	Promega
*Y. lipolytica*		
W29	*MATA, wild* *type*	Barth and Gaillardin [48]
Po1d	*MATA ura3*-*302* *leu2*-*270* *xpr2*-*322*	Barth and Gaillardin [48]
JMY2900 (WT)	Po1d Ura + Leu+	Dulermo et al. [47]
JMY4926	Po1d (Ura- Leu-) + pTEF-riceAlphaAmylase-URA3	This work
JMY5077 (α)	JMY4926 + LEUex	This work
JMY4968	Po1d + pTEF-Glucoamylase-URA3	This work
JMY5083 (GA)	JMY4968 + LEUex	This work
JMY5017 (α + GA)	JMY4926 + pTEF-Glucoamylase-LEU2	This work
JMY3501	*MAT*a *ura3*-*302 leu2*-*270 xpr2*-*322 Δpox1*-*6 Δtgl4* + *pTEF*-*DGA2*-*LEUex* + *pTEF*-*GPD1*-*URAex*	Lazar et al. [41]
JMY3820	*MAT*a *ura3*-*302 leu2*-*270 xpr2*-*322 Δpox1*-*6 Δtgl4* + *pTEF*-*DGA2* + *pTEF*-*GPD1*	Lazar et al. [41]
JMY4930	JMY3820 + pTEF-riceAlphaAmylase-URA3	This work
JMY5035 [JMY3820 (α + GA)]	JMY4930 + pTEF-Glucoamylase-LEU2	This work
JMY5117	JMY5035 (Ura- Leu-)	This work
JMY5196 [JMY3820 (α + GA) × 2]	JMY5117 pTEF-riceAlphaAmylase-URA3 + pTEF-Glucoamylase-LEU2	This work

### Cloning and expression of heterologous amylases

Heterologous genes, alpha-amylase and glucoamylase were synthesized with codon optimization according to the codon usage of *Y. lipolytica* (GenScript) (Additional file 1: Table S1) and cloned in the autocloning vectors of JMP62 type [49, 50] under the control of pTEF promoter. *Bam*HI and *Avr*II were used for the cloning of the genes in the vectors containing either the *LEU2ex* or the *URA3ex* excisable markers (Additional file 5: Figure S3). Expression vectors were digested with *Not*I and subject to gel electrophoresis. The *Not*I bands corresponding to the expression cassettes were extracted from the gel and used for transformation. Expression cassettes were integrated at random in *Y. lipolytica* genome in single copy as described previously [49, 51].

The overexpression cassettes were used for the transformation by the lithium acetate method [52]. Transformants were selected on YNBUra YNBLeu or YNB depending on their genotype. Then genomic DNA from yeast transformants was prepared as described elsewhere [53]. Positive transformants were checked by PCR. Removal of the selection marker was carried out by the LoxP-Cre system widely used in Yarrowia [54].

Restriction enzymes were obtained from OZYME (Saint-Quentin-en-Yvelines, France). PCR amplifications were performed in an Eppendorf 2720 thermal cycler with GoTaq DNA polymerases (Promega). PCR fragments were purified with QIAgen Purification Kit (Qiagen, Hilden, Germany). All the reactions were performed using the manufacturer instructions.

### Protein visualization and enzymatic activity

Supernatant of 6 days culture in YNB were concentrated 10× using Amicon Ultra-0.5 10 K centrifugal filters device (Millipore, Ireland). The concentrated supernatant was loaded into a 10 % SDS-PAGE gel to see the expression and secretion of the heterologous proteins. The molecular weight marker Mark12™ unstained was used as standard (Novex).

Starch-containing plate of YPD was used to determine α-amylase activity. After 3 days of either growing cells or incubating with 5 μl drop from culture supernatant at 28 °C, the plate was stained with iodine vapor. The clear zones around the colonies or the supernatant drops determined the presence of α-amylase activity.

### Growth curves and DCW

To determine DCW in flask experiment, 2 mL of the culture was washed and lyophilized in a pre-weighted tube. The differences in weight correspond to the mg of cells found in 2 mL of culture.

Growth tests were performed in 100 µL cultures in 96-well plates, with constant shaking, in the presence of 1 % soluble starch as the carbon source. Precultures were grown on minimal medium plates, as for the growth tests. Growth was monitored by measuring the optical density (OD_600_ nm) at different intervals, with a microtiter plate reader (Biotek, Colmar, France). For each strain and set of conditions, we used two biological replicates.

### Sugars and citric acid determination

Sugar and citric acid measurement were identified and quantified by HPLC (UltiMate 3000, Dionex-Thermo Fisher Scientific, UK) using an Aminex HPX87H column coupled to UV (210 nm) and RI detectors. The column was eluted with 0.01 N H_2_SO_4_ at room temperature and a flow rate of 0.6 mL min^−1^. Identification and quantification were achieved via comparisons to standards. Before being subject to HPLC analysis, samples were filtered on 0.45-μm pore-size membranes. The quantification of remaining starch was calculated by determining the glucose units present in 200 μL of the media after treated with 1 mL of 2 M HCl and complete hydrolysis was accomplished by heating the mixture in a boiling water bath for 30 min. After neutralization of the hydrolysate with 1 mL of 2 M NaOH, the reducing sugars released from the starch were determined by HPLC.

### Lipid quantification

Lipids from aliquots of 10–20 mg of cells were converted into their methyl esters with freeze-dried cells according to Browse et al. [55] and used for gas chromatography (GC) analysis. GC analysis of FA methyl esters was performed with a Varian 3900 instrument equipped with a flame ionization detector and a Varian FactorFour vf-23 ms column, where the bleed specification at 260 °C is 3 pA (30 m, 0.25 mm, 0.25 μm). FA was identified by comparison to commercial FA methyl ester standards (FAME32; Supelco) and quantified by the internal standard method, involving the addition of 50 μg of commercial C17:0 (Sigma).

### Microscopic analysis

Images were acquired using a Zeiss Axio Imager M2 microscope (Zeiss, Le Pecq, France) with a 100× objective and Zeiss filters 45 and 46 for fluorescent microscopy. Axiovision 4.8 software (Zeiss, Le Pecq, France) was used for image acquisition. Lipid bodies visualization was performed by addition of BodiPy^®^ Lipid Probe (2.5 mg/mL in ethanol; Invitrogen) to the cell suspension (*A*_600_ of 5) and after incubation for 10 min at room temperature.

Electronic microscopy was performed as INRA-AgroParisTech platform Microscopie et Imagerie des Micro-organismes, Animaux et Aliments (MIMA2, Massy, France). The samples were critical point dehydrated (Quorum Technologies K850, Elexience, France) using carbon dioxide as the transition fluid and coated with gold–palladium (272 Ǻ of thickness) in an automatic sputter (Polaron SC7640, Elexience, France). High-magnification imaging of the *Bacillus subtilis* biofilms was performed at an operating voltage of 2 kV under an S-4500 Hitachi FESEM (Hitachi, Japan) at the MIMA2 platform.

### Prediction of biodiesel properties

Mathematical equations and predictive models were used to theoretically determine all the physicochemical fuel-related parameters of the biodiesel obtained by transmethylation of *Y. lipolytica* engineered strains grown in industrial starch. The equations and models used have been previously described in detail by Ledesma-Amaro et al. [56] based on previous theoretical and empirical studies [46, 57–59].

## Additional files

**Additional file 1: Table S1.** Optimized genes used in this work. Targeting sequences are shown: Pre region underlined and X-Ala sequences in bold.
**Additional file 2: Figure S1.**

**Additional file 3: Figure S2.**

**Additional file 4: Table S2.** Biodiesel properties of FAMEs from *Y. lipolytica* CBP from raw starch.
**Additional file 5: Figure S3. **

## Abbreviations

TA: triacylglycerol
GA: glucoamylase
α: α-amylase
DCW: dry cell weight
SEM: scanning electron microscope
