# Supplementary material for: Engineering Yarrowia lipolytica to produce biodiesel from raw starch
Source: Biotechnol Biofuels. 2015 Sep 15;8:148. doi: 10.1186/s13068-015-0335-7 (PMC4571081; doi:10.1186/s13068-015-0335-7)
Supplement: Supplementary file 1 — Additional file 1: Table S1. Optimized genes used in this work. Targeting sequences are shown: Pre region underlined and X-Ala sequences in bold. [file 13068_2015_335_MOESM1_ESM.docx]

**Additional file 1: Table S1: optimized genes used in this work.** Targeting sequences are shown: Pre region underlined and X-Ala sequences in bold.

| Gene name | Sequence |
| --- | --- |
| Alpha amylase from *O. sativa* | ATGAAGCTGTCTACCATTCTGTTTACCGCTTGTGCTACT**CTGGCTCTCGCTCTGGCT**CAGGTTCTGTTTCAGGGTTTCAACTGGGAGTCTTGGAAGGAGAACGGCGGATGGTACAACTTCCTGATGGGAAAGGTCGACGATATCGCCGCTGCCGGTATTACCCACGTCTGGCTGCCCCCTCCCTCTCATTCCGTTGGCGAGCAGGGATACATGCCTGGACGACTGTACGACCTCGATGCCTCGAAGTACGGTAACGAGGCTCAGCTGAAGTCTCTCATTGAGGCCTTCCACGGCAAGGGCGTGCAGGTCATCGCCGACATCGTGATTAACCACCGAACCGCTGAGCATAAGGATGGACGAGGTATCTACTGTCTGTTTGAGGGTGGCACTCCTGACTCCCGACTCGATTGGGGTCCCCACATGATCTGCCGAGACGATCCTTACGGCGACGGAACCGGTAACCCTGACACTGGAGCTGATTTCGCTGCCGCTCCCGACATTGATCATCTGAACAAGCGAGTCCAGCGAGAGCTCATTGGTTGGCTGGACTGGCTCAAGATGGACATCGGCTTCGATGCCTGGCGACTGGACTTTGCTAAGGGATACTCCGCCGACATGGCTAAGATCTACATTGATGCCACCGAGCCTTCGTTTGCCGTTGCTGAGATCTGGACTTCTATGGCCAACGGAGGTGACGGAAAGCCCAACTACGATCAGAACGCTCACCGACAGGAGCTGGTCAACTGGGTTGACCGAGTGGGCGGAGCCAACTCGAACGGCACCGCTTTCGACTTTACCACTAAGGGTATTCTCAACGTGGCTGTGGAGGGAGAGCTGTGGCGACTCCGAGGTGAGGACGGCAAGGCTCCTGGAATGATCGGTTGGTGGCCCGCCAAGGCTACCACTTTCGTTGACAACCACGATACCGGCTCCACTCAGCATCTGTGGCCTTTTCCCTCGGACAAGGTCATGCAGGGCTACGCCTACATTCTCACCCACCCTGGAAACCCCTGTATCTTCTACGACCATTTCTTTGATTGGGGCCTGAAGGAAGAGATCGAGCGACTCGTCTCTATTCGAAACCGACAGGGAATCCACCCTGCTTCTGAGCTGCGAATTATGGAGGCCGACTCCGATCTGTACCTCGCTGAGATCGACGGCAAGGTCATCACTAAGATTGGACCCCGATACGACGTCGAGCATCTGATTCCCGAGGGATTTCAGGTGGTGGCTCACGGAGACGGTTACGCTATTTGGGAGAAGATTTGA |
| Glucoamylase from *A. niger* | ATGAAGCTCTCTACCATCCTGTTCACTGCTTGTGCCACC**CTGGCTCTCGCCCTGGCT**AACGTGATTTCCAAGCGAGCCACCCTCGACTCCTGGCTGTCGAACGAGGCTACTGTGGCCCGAACCGCTATCCTGAACAACATTGGTGCTGACGGAGCTTGGGTCTCCGGAGCTGATTCGGGTATCGTGGTCGCTTCGCCCTCTACCGACAACCCTGATTACTTTTACACTTGGACCCGAGACTCTGGCCTCGTCCTGAAGACCCTCGTTGACCTGTTCCGAAACGGAGATACTTCTCTGCTCTCCACCATCGAGAACTACATTTCCGCCCAGGCTATCGTGCAGGGCATTTCGAACCCCTCTGGAGACCTCTCTTCCGGTGCTGGCCTGGGAGAGCCTAAGTTTAACGTCGATGAGACCGCTTACACTGGTTCTTGGGGCCGACCCCAGCGAGACGGTCCTGCTCTGCGAGCCACCGCTATGATCGGTTTCGGCCAGTGGCTGCTCGACAACGGCTACACTTCTACCGCCACTGATATTGTTTGGCCCCTCGTGCGAAACGACCTGTCCTACGTCGCTCAGTACTGGAACCAGACTGGTTACGACCTCTGGGAGGAAGTGAACGGCTCGTCTTTCTTTACCATTGCTGTGCAGCACCGAGCTCTGGTCGAGGGCTCTGCTTTCGCTACCGCCGTGGGTTCCTCGTGTTCTTGGTGCGACTCCCAGGCCCCCGAGATCCTCTGTTACCTGCAGTCGTTCTGGACCGGCTCTTTTATTCTGGCCAACTTCGACTCTTCCCGATCGGGCAAGGATGCTAACACTCTGCTGGGCTCTATCCACACCTTTGACCCTGAGGCCGCTTGTGACGATTCCACCTTCCAGCCCTGCTCGCCTCGAGCTCTGGCCAACCATAAGGAAGTGGTGGACTCTTTCCGATCTATCTACACCCTCAACGACGGACTGTCCGATTCGGAGGCTGTGGCTGTCGGTCGATACCCTGAGGACACTTACTACAACGGCAACCCTTGGTTCCTCTGCACCCTGGCCGCTGCCGAGCAGCTCTACGACGCCCTGTACCAGTGGGATAAGCAGGGCTCTCTGGAGGTTACCGACGTGTCGCTCGATTTCTTTAAGGCCCTGTACTCTGACGCTGCCACCGGCACTTACTCGTCTTCCTCGTCTACTTACTCCTCGATCGTCGACGCCGTTAAGACCTTCGCTGATGGATTTGTGTCTATTGTCGAGACCCATGCTGCCTCCAACGGATCTATGTCCGAGCAGTACGACAAGTCTGATGGAGAGCAGCTCTCCGCCCGAGACCTGACTTGGTCTTACGCTGCCCTGCTCACCGCTAACAACCGACGAAACTCCGTCGTTCCCGCCTCGTGGGGAGAGACCTCTGCTTCTTCCGTGCCTGGTACTTGTGCTGCCACCTCCGCCATCGGCACCTACTCGTCTGTTACCGTGACTTCTTGGCCCTCCATTGTTGCCACCGGCGGAACCACTACCACTGCTACCCCTACTGGATCGGGTTCTGTCACCTCCACTTCGAAGACCACTGCTACCGCCTCTAAGACCTCTACTTCCACCTCCTCGACTTCCTGCACCACTCCCACCGCTGTCGCCGTTACTTTCGACCTGACCGCCACCACTACCTACGGAGAGAACATCTACCTCGTGGGTTCGATTTCTCAGCTGGGCGACTGGGAGACTTCGGATGGTATCGCCCTGTCTGCTGACAAGTACACCTCTTCCGATCCCCTCTGGTACGTGACTGTCACCCTGCCTGCCGGCGAGTCTTTCGAGTACAAGTTTATCCGAATTGAGTCGGACGATTCTGTCGAGTGGGAGTCTGATCCCAACCGAGAGTACACCGTTCCTCAGGCCTGCGGAACTTCCACCGCTACTGTGACCGACACTTGGCGATAG |
