# Supplementary figures and images for: Engineering Yarrowia lipolytica to produce biodiesel from raw starch
Source: Biotechnol Biofuels. 2015 Sep 15;8:148. doi: 10.1186/s13068-015-0335-7 (PMC4571081; doi:10.1186/s13068-015-0335-7)

## Additional file 2: Figure S1

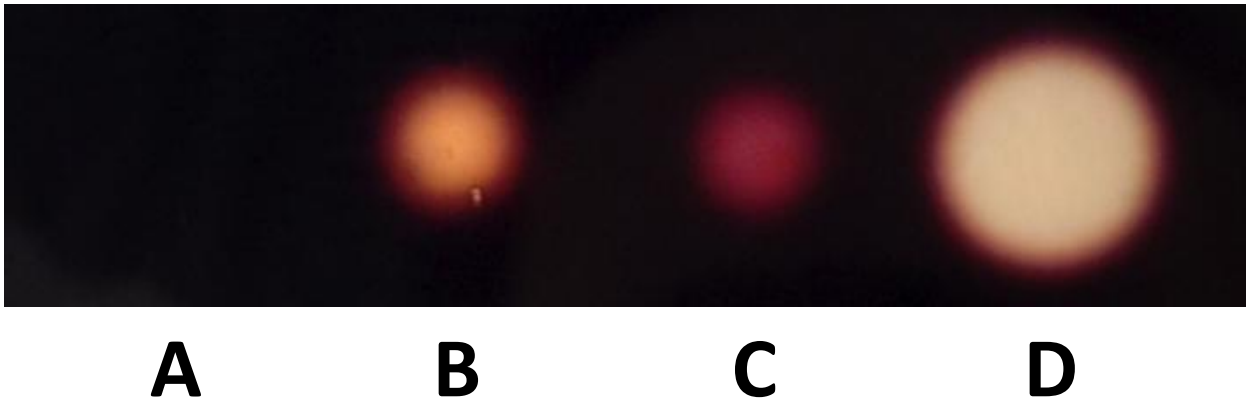

Supplement: Supplementary file 2 — Additional file 2: Figure S1. [file 13068_2015_335_MOESM2_ESM.pdf]

# Additional file 3: Figure S2

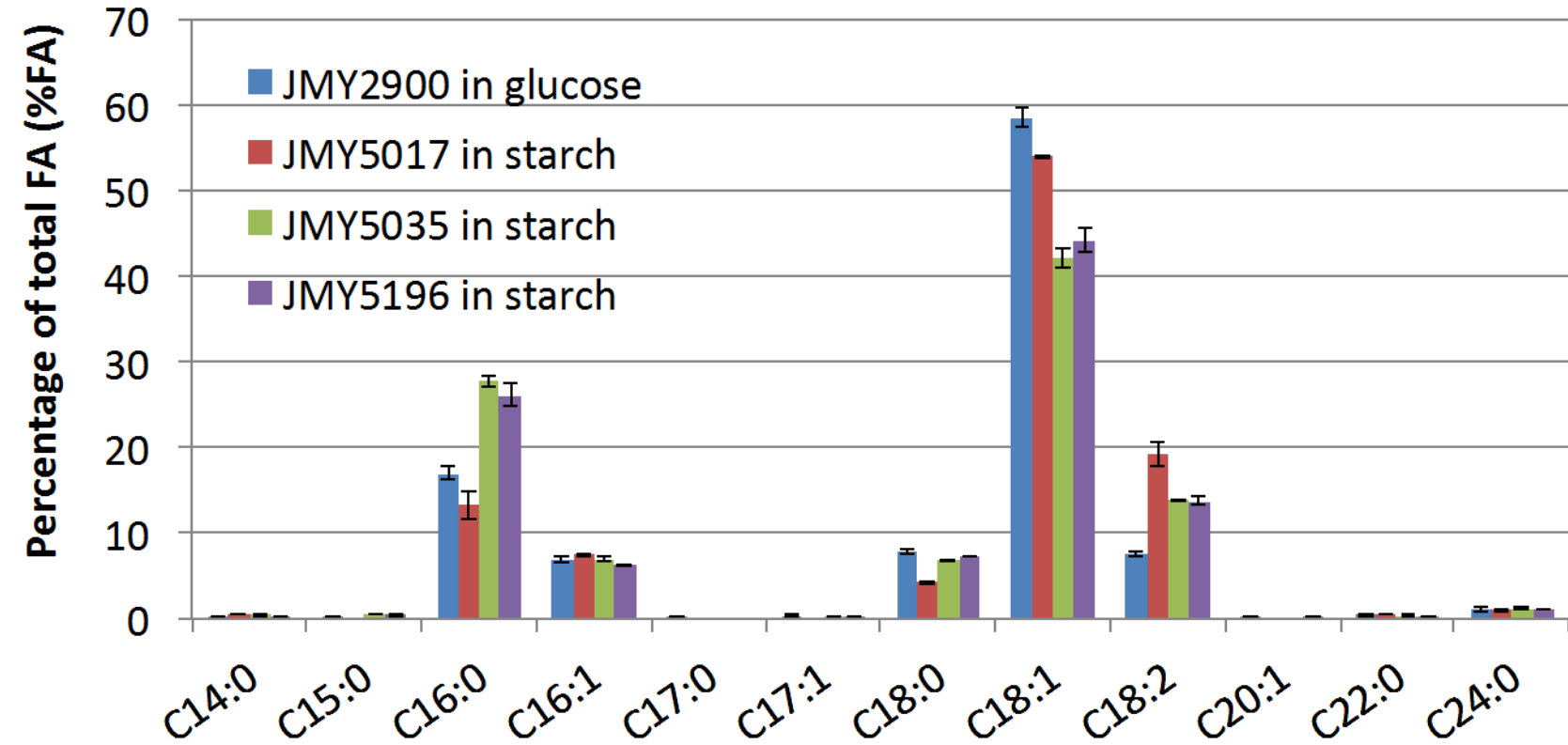

Supplement: Supplementary file 3 — Additional file 3: Figure S2. [file 13068_2015_335_MOESM3_ESM.pdf]

# Additional file 5: Figure S3

**A**

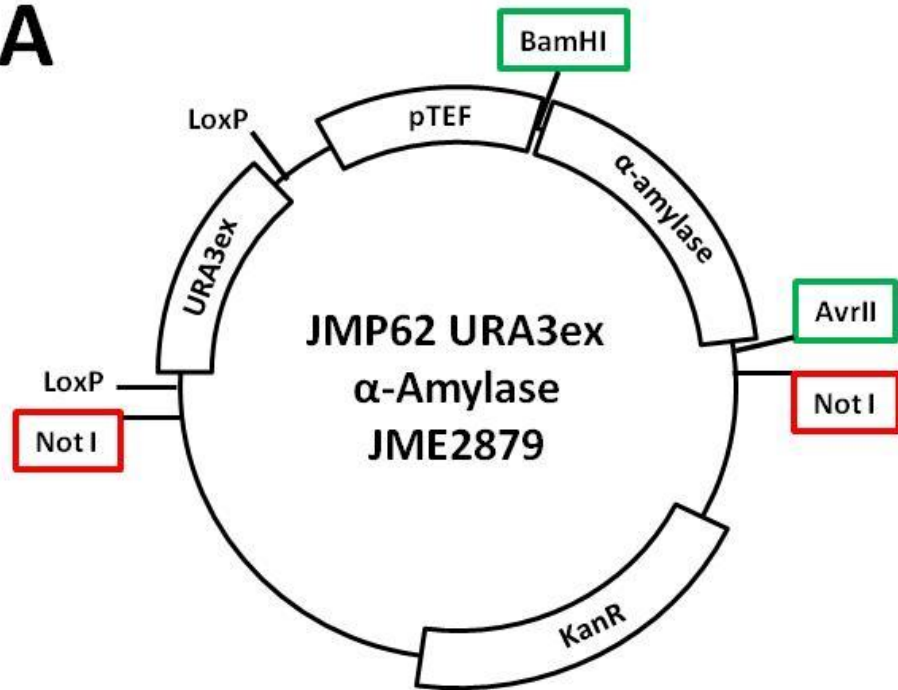

**B**

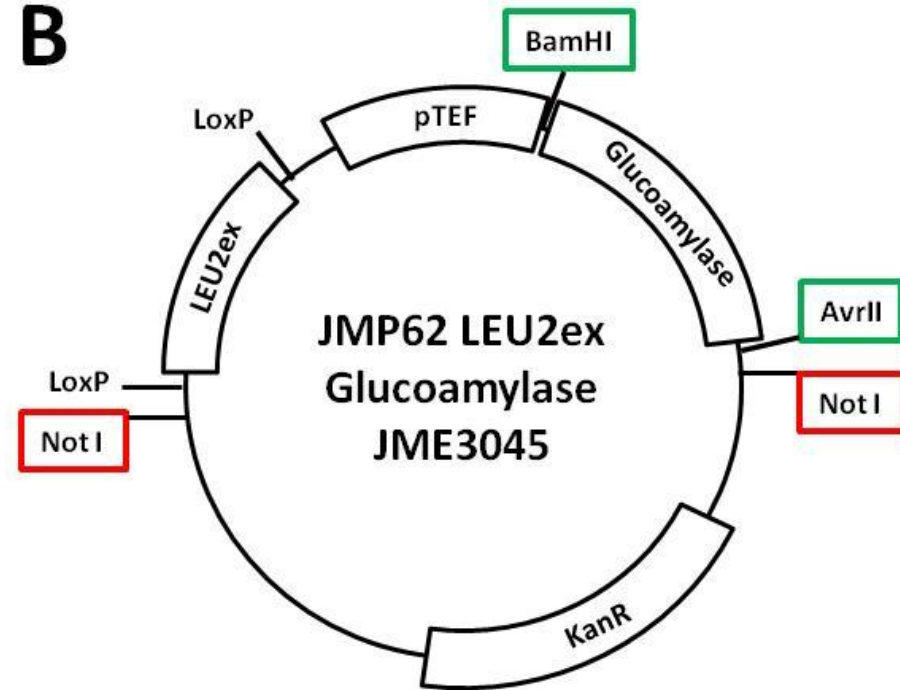

Supplement: Supplementary file 5 — Additional file 5: Figure S3. [file 13068_2015_335_MOESM5_ESM.pdf]
