# Supplementary material for: Engineering Yarrowia lipolytica to produce biodiesel from raw starch
Source: Biotechnol Biofuels. 2015 Sep 15;8:148. doi: 10.1186/s13068-015-0335-7 (PMC4571081; doi:10.1186/s13068-015-0335-7)
Supplement: Supplementary file 4 — Additional file 4: Table S2. Biodiesel properties of FAMEs from Y. lipolytica CBP from raw starch. [file 13068_2015_335_MOESM4_ESM.docx]

**Additional file 4: Table S2: Biodiesel properties of FAMEs from *Y. lipolytica* CBP from raw starch**

| **Parameter** | **Units** | **Limits** | | **Strain** | | | |
| --- | --- | --- | --- | --- | --- | --- | --- |
|  |  | Min | Max | JMY2900  in glucose | JMY5017  in raw starch | JMY5035  In raw starch | JMY5196  In raw starch |
| Density 15ºC | g/cm^3^ | 0.860 | 0.900 | 0.872 | 0.873 | 0.867 | 0.868 |
| Kinematic viscosity 40ºC | mm^2^/s | 3.5 | 5 | 4.6 | 4.4 | 4.5 | 4.5 |
| CN | - | 51 | - | 61.0 | 56.7 | 61.1 | 61.2 |
| SN | - | - | - | 187.4 | 176.3 | 184.2 | 184.2 |
| Iodine Value | gI_2_/100g | - | 120 | 69.0 | 88.5 | 66.7 | 66.8 |
| HHV | MJ/kg | - | - | 40.7 | 40.9 | 40.9 | 40.9 |
| CFPP | ºC | - | * | 10.6 | 2.67 | 12.0 | 12.4 |
| Linolenic acid content | Wt% | - | 12 | ND | ND | ND | ND |
| 4 or more double bonds | ºC | - | 1 | ND | ND | ND | ND |

* Not specified in the EN14214 standard. The CFFP is established according to national regulations.
